# Complications of Mandibular Distraction Osteogenesis in Infants with Isolated Robin Sequence

**DOI:** 10.3390/children10101591

**Published:** 2023-09-23

**Authors:** Zhe Mao, Gabriel Tian, Mayank Shrivastava, Jiawei Zhou, Liang Ye

**Affiliations:** 1 Department of Oral and Maxillofacial Surgery, Guangzhou Women and Children’s Medical Center, Guangzhou 510000, China; maozhe19840723@gwcmc.org (Z.M.);; 2 Department of Rehabilitation Medicine, Medical School, University of Minnesota, Minneapolis, MN 55455, USA; 3 Orofacial Pain, TMD & Dental Sleep Medicine at Adams School of Dentistry, University of North Carolina at Chapel Hill, Chapel Hill, NC 27599, USA; shriv20@unc.edu

**Keywords:** Robin Sequence, infant, MDO, complication

## Abstract

Mandibular Distraction Osteogenesis (MDO) is now the preferred procedure to alleviate airway obstruction in infants with severe Robin Sequence (RS). However, there have been very few studies investigating complications related to MDO surgery performed on patients affected by isolated RS. In this study, age at distraction, weight at distraction, preoperative intubation, repeat MDO and complications associated with MDO were included as variables. Minor, moderate and major problems were evaluated and recorded as surgical site infections (SSI), injuries to the facial nerve, self-extinction hypertrophic scars, temporomandibular joint ankylosis, device failures, early ossification and fibrous non-union. One hundred and fifty one patients with isolated RS were included. At distraction, the mean age was 72 days (12–540 days) and the mean weight was 4.05 kg (2.4–12.2 kg). Only one patient needed tracheostomy after MDO, and none required further distraction. Ultimately, the complication rate was 15.23%, and there was a total of 7.95% minor, 9.27% moderate and 0% major complications. Minor incidents included surgical site infection (SSI) managed with antibiotics taken orally (*n* = 8), neuropraxia in the VII cranial nerve (CN) (*n* = 1), and hypertrophic scarring (*n* = 3). Incidents reported as moderate were SSIs managed with intravenous antibiotics (*n* = 9), incision and drainage (*n* = 3) and self-extubation (*n* = 2). There was no case of TMJ ankylosis. There were no cases of early or premature ossification, fibrous non-union and device fracture. In conclusion, MDO is an effective and appropriate management technique for infants with isolated RS and severe airway obstruction. Infections at the surgery site accounted for the vast majority of the complications. Further investigations may be needed to determine the long-term consequences of MDO.

## 1. Introduction

Robin Sequence (RS) is a congenital condition characterized clinically by micro-gnathia, glossoptosis and upper airway obstruction. The incidence of RS ranges from 1 in 8500 to 14,000 people [1,2,3]. This heterogenic entity can occur in isolation or as part of a syndromic pattern of symptoms. The condition occurs alone in 20–50% of cases [2,3,4]. Isolated RS has been linked to KCNJ2 dysregulation or DNA alterations near the SOX9 gene [2,5,6]. Non-genetic factors, such as pregnancy conditions that limit jaw growth, can also induce isolated RS. Syndromic RS is genotypically variable, as is syndromic Stickler which correlates with changes in the collagen genes and velocardiofacial syndrome from a 22q 11.2 microdeletion [7].

Nonsurgical treatment to assist with breathing includes positioning, nasopharyngeal airway and Tübingen Palatal Plate treatments. When one suffers from severe airway obstruction, which cannot be addressed by nonsurgical treatment, a surgical treatment such as Mandibular Distraction Osteogenesis (MDO) is needed. MDO can reduce airway blockage by lengthening the mandible and stretching tongue attachments to it [8,9,10]. MDO is now serving as the preferable modulation to address airway obstruction in severe RS patients [10,11,12,13,14,15]. However, there have been a few studies that have investigated the complications associated with MDO.

The phenotypes of syndromic RS phenotypes vary considerably in terms of the craniofacial airway, neurological abnormalities, the immune system and cardio-vascular abnormalities. This emphasizes various considerations that must be taken into account during surgery planning as well as complications and morbidity solutions [16,17,18]. For syndromic RS specifically, genetic syndromes frequently presenting alongside RS may lead to increased complication rates and/or morbidity in affected patients. Previous studies evaluating complications pertaining to the MDO procedure for RS included a mixed group of syndromic and non-syndromic patients [19,20,21,22,23,24,25]. Investigating only isolated RS prevents these syndromic variables and builds a more moderated model of mandibular distraction complications. Unlike previously reported studies, our work can investigate isolated RS independently given that we have a reasonably large sample, which may result in the improved accuracy of the results and conclusions. The objective of this study was to investigate MDO-related complications in infants with isolated RS. This is the first study to investigate complications of MDO only in patients with isolated RS.

## 2. Materials and Methods

In this retrospective study, an assessment of complications associated with MDO was performed. These complications included surgical site infection (SSI), self-extubation, hypertrophic scarring, temporomandibular joint (TMJ) ankylosis, device fracture, fibrous nonunion, death, premature ossification and craniofacial nerve injury. The classification of these complications followed Davidson et al.’s definitions of minor, moderate and major complications [26]. Complications that were minor were defined as incidents that did not result in negative outcomes and could be managed with non-invasive care [19,26]. Moderate complications were events which could have led to adverse outcomes but which could also be managed with invasive procedures [19,26]. Major complications were those resulting in adverse outcomes unresolvable with invasive therapy [19,26].

### 2.1. Sample Inclusion

The study was given approval by the Ethics Committee at the institution. A clinical consensus report was used to diagnose RS. Every single case was diagnosed by two doctors. The initial diagnosis of RS was made in patients who had micrognathia, glossoptosis and airway obstruction. [1]. Glossoptosis and airway obstruction were further confirmed in all patients with a fiberoptic nasopharyngoscopy examination, resulting in the final diagnosis [1,27,28]. Amongst patients who were included in the study, a single level of tongue-based airway obstruction did not respond well to nonsurgical interventions. The exclusion criteria were severe cardiopulmonary diseases, head and neck tumors or trauma, central apnea or mixed apnea, laryngomalacia, syndromic RS and other anomalies causing airway obstruction. 

### 2.2. Surgical Protocol

As previously described, all included patients were treated with MDO [14,15]. CBCT images were used to determine the osteotomy line. A submandibular incision was made 1.5 cm below and parallel to the inferior border of the mandible. To avoid the tooth bud and condylar neck, a “walking-stick”-shaped osteotomy line that extended to the mandible angle was used. Patients were subjected to mandibular distraction at a rate of 1.2 mm per day. Until symmetrical alignment of the upper and lower jaws, or a slight underbite was established, distraction was performed. The mean duration of the distraction process was 10 days.

### 2.3. Statistics

SPSS version 19 was used for statistical analysis. Descriptive statistics were recorded for all included patients. Complications associated with the MDO were categorized as minor, moderate and major [19,26]. Values of gestational age, age at distraction, weight at distraction and follow-up duration were reported as mean (range).

## 3. Results 

**Figure 1:**
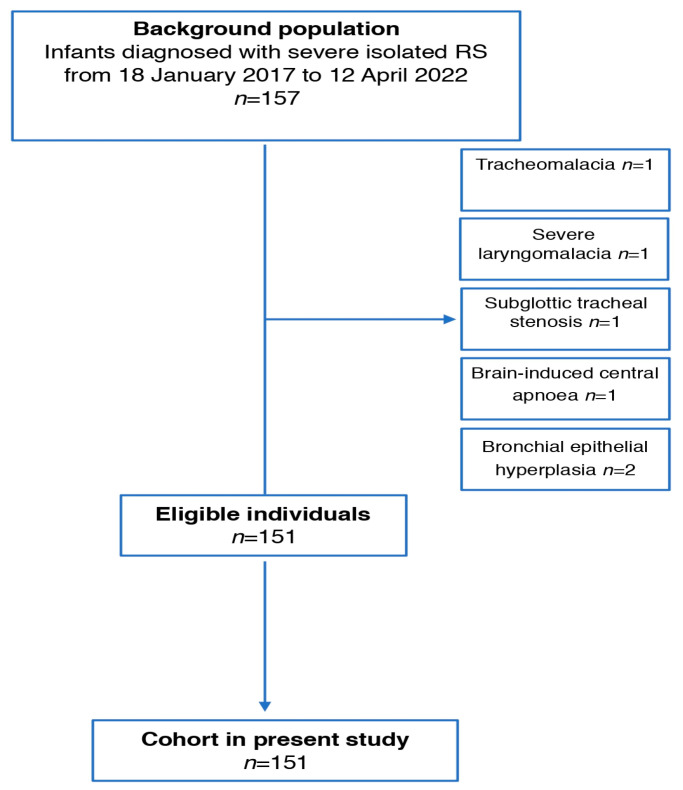
Flow chart of patients recruited in the study.

There were 157 infants diagnosed as having isolated RS over the study period (18 January 2017 to 12 April 2022) and 157 infants were diagnosed with isolated RS (Figure 1). Of these patients, 151 met the inclusion criteria (Figure 1). In excluded patients, one had tracheomalacia, one had severe laryngomalacia, one had subglottic tracheal stenosis, one had brain-induced central apnea and two had bronchial epithelial hyperplasia.

The mean age of starting MDO in 151 patients was 2.4 months, the mean gestational age was 38 weeks and the mean birth weight was 4.05 Kg (Table 1). A total of 17.5% of included patients had low birth weight, 15.3% premature birth, 87% cleft palate, 25.8% preoperative intubation, 2.6% lower airway abnormalities and 0.7% gastroesophageal reflux disease (GERD) (Table 1). None received a gastrostomy tube or Nissen fundoplication (Table 1).

**Figure 2:**
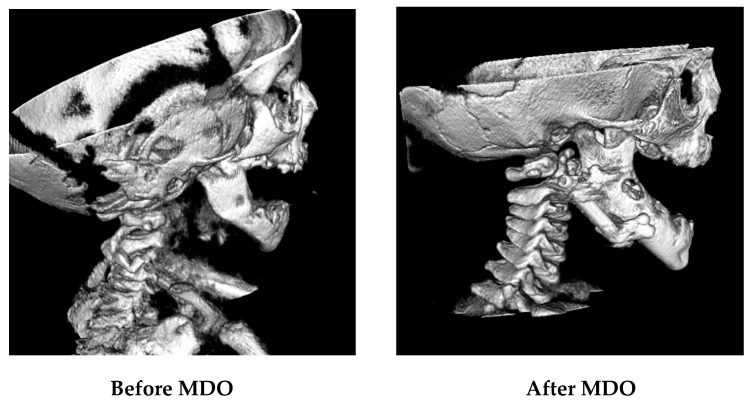
Hard tissue (bone) in CBCT images before and after MDO.

**Figure 3:**
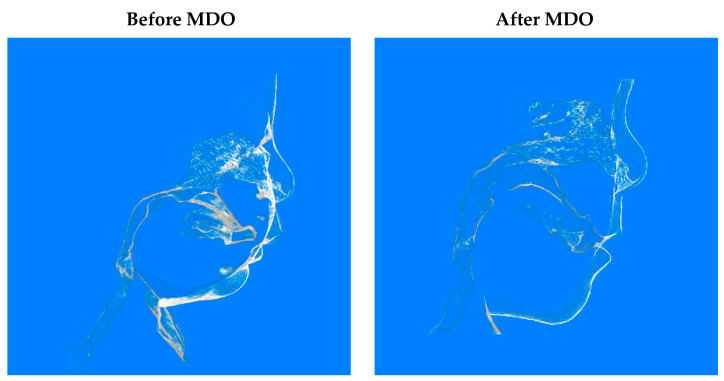
Soft tissue in CBCT images before and after MDO.

Airway surgical success was achieved in 99.3% of included patients. One patient required a tracheostomy after MDO. There were no patients who needed repeat distraction. Micrognathia was corrected (Figure 2), and later, the profile was improved (Figure 3).

**Figure 4:**
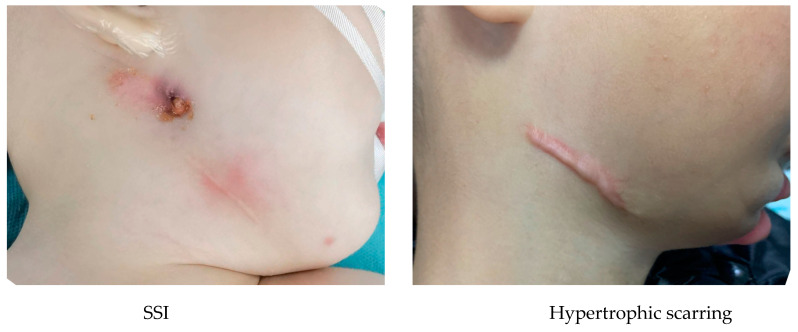
Complications (SSI and hypertrophic scarring).

There were no mortalities. Complications were reported at a rate of 15.23%. These incidences included 7.95% minor, 9.27% moderate, and 0% major complications (Table 2). The minor incidents were surgical site infection (SSI) managed with antibiotics taken orally (*n* = 8), neuropraxia of VII cranial nerve (*n* = 1), and hypertrophic scarring (*n* = 3). The vast majority of the complications were infections at the surgery site. SSI managed with intravenous antibiotics (*n* = 9), SSI managed by the incision and drainage of an abscess (*n* = 3), and self-extubation (*n* = 2) were moderate events. All SSI patients managed with incision and drainage demonstrated hypertrophic scarring (Figure 4). There were no cases of ankylosis of TMJ and brous non-union, early or premature ossi cation and single- device fracture.

## 4. Discussion

Upon severe airway obstruction and unsuccessful non-invasive therapy, surgical interventions such as MDO are considered. The objective of this study was to investigate MDO-related complications in infants with isolated RS. 

MDO was first put into practice by McCarthy et al. [29], leading to a steadily increasing utilization of the technique on patients with RS for the purpose of avoiding tracheostomy. Whilst complications of the mandibular distraction procedure have gained interest over the last few years, many studies focusing on the complications associated with this procedure on patients afflicted by RS have been limited to small sample sizes in the single to double digits [19,20,21,22,23,24,25], creating lower statistical significance across data collected. In syndromic RS, genetic syndromes presenting alongside RS such as Treacher Collins syndrome, Stickler syndrome and the micro-deletion of 22q 11.2 may cause a poor immune system [30], additional facial deformation [18] and heart defects [7,31]. These could lead to increased complication rates and/or morbidity in affected patients [7,31]. Investigating only isolated RS prevents these syndromic variables and builds a more moderated model of mandibular distraction complications. This is the first study to investigate MDO-related complications specifically in infants with isolated RS.

Our study aimed to investigate MDO complications in infants with isolated RS specifically on a greater sample size than was ever previously investigated in a single study. In terms of our outcomes, we recorded various cases of SSI including those treated by oral antibiotics (5.30%), those treated by intravenous antibiotics (5.96%) and those requiring incision and drainage (1.99%), CN VII neuropraxia (0.66%), hypertrophic scarring (1.99%), and self-extubation (1.32%). Amongst these complications, 7.95% were minor and 9.27% were moderate. There were no major complications recorded. Amongst these results, SSI was the most common, making up 13.25% of the total sample population and 76.92% of all complications. In a study by Murage et al., they found rates of SSIs treated with oral antibiotics incidence were 12%, intravenous antibiotics incidence 8%, abscess incision and drainage incidence 2%, single-device fracture incidence 2%, self-extubation incidence 4% and CN VII neuropraxia incidence 2%. None of them demonstrated TMJ ankylosis, premature ossification, revisions of hypertrophic scars or fibrous nonunion. Overall, their study had a minor complication rate of 14%, moderate complication rate of 16% and a major complication rate of 0%. Murage et al.’s study compiles other signicant studies’ complication rates, noting that their average complication rates were 9% for major, 15.10% for moderate, and 15% for minor [19] (Table 3). Compared to these results, our study shows a lower complication frequency. This could possibly be attributed to the unique usage of isolated RS patients, as opposed to the primarily mixed cohorts, including both isolated and syndromic RS in Murage et al. and others’ studies, which controls for additional co-morbidities that may cause more complications. Additionally, our study had a higher mean birth weight of 4.05 kg vs. 2.98 kg, which could be another reason why our compilation rate was lower overall [32].

Most reports discussing complications of mandibular distraction for RS outline additional complications for RS cases associated with another genetic syndrome. In a retrospective review by Ali-Khan et al., concerning additional impediments of Treacher Collins Syndrome versus regular RS, successful decannulation within a year after primary distraction was significantly lower in frequency for syndromic association (21%), compared to solely RS (65%). There was also a higher rate of repeat distraction amongst syndromic cases [31] (Table 3). Breik et al. report considerably lower success rate in preventing tracheostomy, relieving upper airway obstruction and tracheostomy decannulation in syndromic populations, being four times as likely to fail in these aspects compared to those with isolated RS [33]. Although end surgical success was found not to be statistically significant, Soto et al. observed that the total number of distraction attempts was significantly higher than expected in syndromic groups. The research group also acknowledges that syndromic RS patients are more complex than isolated RS patients, potentially requiring secondary interventions to alleviate airway and feeding obstructions [12]. Breik et al. observed that RS cases with associated syndromes following mandibular distraction had a 5 times higher risk of requiring feeding adjuncts after MDO [34]. In light of these additional complications and uncertainties, it should be noted that much of the literature data on the difference between syndromic and isolated RS mandibular distraction cases are specific to a genetic anomaly coupled with the symptoms of RS. In our study, only cases of isolated RS were recruited; syndromic RS cases were excluded.

The effects of mandibular distraction treatment timing on cases with RS have been explored. In 2011, Kolstad et al. reviewed data from a 9-year period, observing that patients treated when older (>5 months) have fewer overall complications than patients treated when younger (≤5 months) [35]. Longer timeframe data comparison in a 2014 study by Murage et al. reported that MDO is possibly safer in infants compared to adults or older children [19]. The average age at the time of MDO in our study was 2.4 months. Breik et al. identified findings suggesting that different distraction rates are optimal for complication reduction in external mandibular distraction procedures at different treatment ages. Higher ages, indicated by a lower distraction rate than lower ages (1 mm/d vs. 2 mm/d), had greater incidences of technical failures, a finding hypothesized by Breik et al. to have been influenced by increased movement and thus the likelihood of distractor disturbance [36]. In more recent 2020 studies by Lee et al. and Ramirez-Garcia et al., neonatal intervention (treatment at <28 days of age) was investigated [20,37]. Neonatal intervention was found to have no statistical difference in Lee et al.’s study, even being more successful with fewer complications compared to older patients between 1 month and 2 years of age in Ramirez-Garcia et al.’s study [20,37]. It is important to note that the quantification techniques each of these studies used in relation to their assessment of treatment complication frequency greatly differ, and may not be adequate to assess significant statistical correlations. For example, Lee et al.’s study used an indirect measurement of complication frequency in the form of treatment costs, whilst Kolstad et al. directly used previously gathered patient data [20,35]. Further statistical exploration and review of the effect of treatment age on mandibular distraction complications in individuals with RS should be considered.

Distractor devices can be external or internal and non-resorbable or resorbable. A preference for internal distraction devices is quite clear amongst much of the available literature on complications of mandibular distraction due to the overall lowered chance of device failure [38,39] (Wittenborn et al., Mandell et al.), simplified distraction and consolidation care [38,39] (Wittenborn et al., Mandell et al.) and more relaxed psychosocial effects for both the patient and the patient’s parents [39] (Mandell et al.). Whilst Genecov et al. outline that external distractors perform be er when large advancements, multidirectional vectors, and the presence of adequate bone stock volume are needed, external devices have been described as having a greater incidence of major complications [40] (Table 3); Shetye et al. reported complications of TMJ ankylosis and degenerative changes with the higher incidence in patients undergoing external distraction of grafted bone (5.6%), with internal buried devices having a lower major complication frequency [20]. According to Paes et al., non-resorbable internal and external devices may be susceptible to breakage requiring secondary operation as opposed to resorbable devices [41]. Whilst also minimizing additional surgical removal and scar formation [22], internal resorbable devices were found to have a higher rate of failure by dislodgement or instability [40]. It is important to consider that Paes et al. found hypertrophic scarring was only seen in external distractors, whilst we had three cases in our study using internal distractors [41]. In our study, only internal, non-resorbable distractors were used in patients affected by isolated RS.

According to Master et al., the complications from MDO included relapse in 64.8%, tooth injury in 22.5%, hypertrophic scarring in 15.6%, nerve injury in 11.4%, infection in 9.5%, inappropriate distraction vector in 8.8%, device failure in 7.9%, fusion error in 2.4% and temporomandibular joint injury 0.7% [23]. Amongst these issues, major events such as temporomandibular joint injury, fusion error, and device failure were classified as major events. As mentioned, with a similar classification system, Shetye et al. found a much higher incidence of TMJ ankylosis (5.6%) as compared to Master et al.’s study (0.7%) [21]. However, the two studies had similar fusion error rates of 2.44% and 2.4% for Shetye et al. and Master et al., respectively [21,23]. Tahiri et al. noted fibrous non-union rates of 1.2% in lower-weight infants compared to the two previous studies, suggesting a difference between major complication rates of different weight classes of RS-afflicted infants subjected to mandibular distraction [32] (Table 3). The device malfunction rate of Master et al. is further corroborated by Manrique et al., who found 7.9% of patients had a distractor breakage, which required surgical replacement in phases that were not consolidatory [42] (Table 3). However, these studies were not specifically focused on MDO managing isolated RS.

Neuropathic pain can happen because of a disease or lesion involving the somatosensory system [43,44,45,46,47,48]. Neuropathic pain in the orofacial region can be the clinical manifestation following oral surgeries or injuries [49,50,51,52,53]. Neuropathic pain has been a secondary focus of many studies in favor of more immediately detectable complications of mandibular distraction on infants afflicted by RS, such as minor infection. Long-term developments, particularly in the facial alveolar nerves, have yet to be considered alongside immediate complications. In a study by Steinberg et al., inferior alveolar nerve injury is rare (2.5%); however, permanent lower lip depressor weakness is more common compared to previous studies (15% of sides) [24] (Table 3). Furthermore, Chocron et al. observed nerve injuries in 3.2% of patients as a complication of mandibular distraction in RS populations. In terms of alveolar nerve injury frequency (0.08%), Chocron et al. speculate that the incidence rate is likely higher as the majority of studies did not carry out objective assessment for nerve injuries [25]. In a survey sent to surgeons about their experience with FND in patients, Crowder et al. determined the majority of respondent surgeons (63.8%) observed FND as a complication associated with mandibular distraction, with a total of 17 (21.3%) surgeons reporting experience with permanent FND [54]. This study is limited by the exposure of different surgeons to patients, as a surgeon more frequently exposed to FND cannot be detected in the data pool. Additional studies by Zellner et al., Tibersar et al., Allam et al. and Mudd et al. all found incidences of FND at hovering around 10% of distraction patients (10%, 9%, 11% and 8.4%, respectively), suggesting important consideration for such complications before MDO treatments in patients with RS [55,56,57,58].

**Table 3:** Comparison of studies reporting complications of mandibular distraction in patients with Robin Sequence.

Year	Author	*n*	Placement	Type of Distractor	Complications			
					Total	Minor	Moderate	Major
2014	Murage et al. [19]	50	Internal	Nonresorbable	30%	14%	16%	0%
2016	Steinberg et al. [24]	44	Internal	Nonresorbable				TMJ Ankylosis 2.27% (*n* = 1)
2010	Davidson et al. [26]	211	Both Semi Buried (21.3%) External (61.1%)	Nonresorbable		Semi Buried (62%) External (26%)	Semi Buried (18%) External (22%)	Semi Buried (0%) External (unspecified)
2018	Ali-Khan et al. [31]	24			67%			20%
2015	Tahiri et al. [32]	121 <4 kg (66.9%) >4 kg (33.1%)			<4 kg (17.3%) >4 kg (25.0%)	<4 kg (9.9%) >4 kg (20.0%)		
2009	Genecov et al. [40]	67	Both	Both	35.4%	22.20%	13.2%	
2021	Manrique et al. [42]	19			10.5%			

Although RS is associated with variety of clinical features, the most significant one is respiratory insufficiency from upper airway obstruction. Nasopharyngeal airway stent, prone positioning, CPAP and nasogastric feeding are non-surgical management techniques of RS, whilst surgical procedures included TLA, tracheostomy and MDO [59]. Several studies have documented the safe and effective use of MDO in treating the upper airway obstruction of RS patients with micrognathia specifically in the neonatal and early infancy period [25]. A meta-analysis reported the effectiveness of MDO in treating upper airway obstruction and also showed success in preventing tracheostomy in 91% of neonates and in relieving symptoms of OSA in 97% of children [33,60]. In a comprehensive literature search with studies from 1981 to June 2015, MDO was associated with a lower percentage of significant airway obstruction post-procedure (3.6%), compared to infants who underwent TLA (50%) [61]. In the past few years, MDO has gradually become the first choice of many surgeons for the treatment of tongue-based airway obstruction in children with PRS [8]. In another study, the effectiveness of MDO for the treatment of upper airway obstruction in children younger than 18 years with mandibular hypoplasia was assessed. The diagnosed cases of PRS, PRS with syndrome and Treacher Collins syndrome were included. MDO was effective in treating airway obstruction in 89% of cases. Success in this study was defined by the avoidance of tracheostomy or CPAP or the significant improvement of OSA symptoms [62]. In another study, airway patency variations, clinical symptoms of airway obstruction and polysomonographic parameters were assessed in children with RS who underwent MDO. In the study, endoscopic scores of airway patency, clinical symptoms of respiratory distress and PSG parameters such as the apnea–hypopnea index, total sleep time and the oxygen desaturation index significantly improved in patients with RS after MDO (<0.05) [63]. Similarly, a prospective study investigated the feeding outcomes and airway of patients managed with MDO, tongue–lip adhesion and conservative management. The study reported that MDO resulted in the greatest decrease in the apnea–hypopnea index followed by tongue–lip adhesion and conservative management. This result was expected since MDO causes the rapid expansion of the pharyngeal airway, whereas tongue–lip adhesion addresses glossoptosis. However, patients with PRS whose airways are stable can have good outcomes with conservative management alone [64]. Furthermore, the MDO complications in pediatric craniofacial centers are rare and peri-operative mortality from the procedure is extremely uncommon. Children with multi-system anomalies are more likely to experience MDO failure, and these children should be evaluated thoroughly and have any other anomalies fixed before MDO.

In particular, comparatively non-invasive procedures such as the Tübingen Palatal Plate treatment (TPP), whilst an effective treatment in resolving airway obstruction in patients with isolated RS, are limited in their ability to achieve the desired facial profile [65,66]. Additionally, 14.3% of patients treated with TPP had incompetent lip closure, and 13.3% of patients snored in comparison to none in the control [66]. TPP has been shown to be more effective in preventing post-treatment tracheostomies in isolated RS compared to in syndromic RS, with a rate of 0% compared to 17% [65]. This isolated RS rate is slightly lower than the rate of post-operative tracheostomy found in this study, which was 0.7%. Additionally, TPP may prevent the additional complications associated with MDO, such as SSI or hypertrophic scarring [66]. There are currently no studies on complications of TPP during treatment. Whilst studies concerning TPP have determined minimal long-term complications, a comparison may not be drawn due to a paucity in research on long-term MDO complications [66]. Lastly, it is important to consider each treatment method relative to each patient’s unique circumstances, as TPP has been found to have a lower cost and burden of care, but to be less successful for more severe cases [65]. Since the recruited patients in our study suffered severe airway obstruction, the surgical modality is the better solution to address the problem.

This study has limitations. The study is not long-term enough to take into account the effects mandibular distraction has on events such as tooth eruption, mandibular growth or further complications related to prolonged development in the orofacial region. 

In conclusion, MDO is an effective and appropriate management for neonates with isolated RS and severe airway obstruction. Peri-operative mortality due to procedure is rare in our center. The most common incidence was SSI, and those that were successfully treated with intravenous antibiotics counted for the majority. Further investigations may be required to determine the long-term effects of MDO. This study represents the first attempt at investigating complications of MDO only in patients with isolated RS.

## Figures and Tables

**Table 1:** Patient demographics.

Characteristics	Value
Number of patients recruited	151
Isolated RS/Syndromic RS	151/0
Post-operative tracheostomy	0.7%
Low birth weight (<2.5 kg)	17.5%
Weight (range), kg	4.05 (2.4–12.2)
Premature (<37 wk)	15.3%
Gestational age (range in week)	38 (35–41)
Age at distraction (range in month)	2.4 (0.3–18)
Mean follow-up (range in month)	15 (8–25)
Cleft palate	131/151 (87%)
Lower airway abnormalities	4 (2.6%)
GERD	1 (0.7%)
Gastrostomy tube	0
Nissen	0
Late operation	1 (0.7%)
Preoperative intubation	39 (25.8%)
Repeat distraction	0

**Table 2:** Post-operative complications.

Complications	*n*	Rate, %
Minor	12	7.95
SSI (managed with oral antibiotics)	8	5.30
CN VII neuropraxia	1	0.66
Hypertrophic scarring	3	1.99
Moderate	14	9.27
SSI (managed with intravenous antibiotics)	9	5.96
SSI Infection (managed with incision and drainage)	3	1.99
Self-extubation	2	1.32
Major	0	0
Total	23	15.23%

## Data Availability

Not applicable.
